# How Do Infants Disaggregate Referential and Affective Pitch?

**DOI:** 10.3389/fpsyg.2018.02093

**Published:** 2018-10-31

**Authors:** René Kager

**Affiliations:** Utrecht Institute of Linguistics OTS, Utrecht University, Utrecht, Netherlands

**Keywords:** infant speech perception, pitch processing, infant language representation, lexical tone acquisition1, Lexical tone perception

## Abstract

Infants are faced with a challenge of disaggregating functions of pitch in the ambient language into affective, pragmatic or referential (the latter in tone languages only). This mini review discusses several factors that might facilitate the disaggregation of referential and affective pitch in infancy: acoustic characteristics of infant-directed speech, recognition of vocal affect, facial cues accompanying affective prosody, and lateralization of affective and referential prosody in the brain. It proposes two hypotheses concerning the role of audiovisual cues and brain lateralization

This article discusses potential factors to facilitate the disaggregation of referential and affective pitch in infancy: acoustic characteristics of infant-directed speech, recognition of vocal affect, facial cues accompanying affective prosody, and lateralization of affective and referential prosody in the brain. It proposes two hypotheses concerning the role of audiovisual cues and brain lateralization.

Among the many acoustic cues in speech, fundamental frequency (perceived as pitch) is arguably the one that, cross-linguistically, has the widest range of linguistic and para-linguistic uses (Gussenhoven, 2004). Universally, pitch signals *affective* use (for example, express happiness by high average pitch and wide pitch range) and *pragmatic* use (for example, marking a question by rising pitch is a universal tendency). Exclusively in tone languages, pitch supports *referential* use by contrasting word meanings (for example, Cantonese /fan/“divide” carries a high-level tone; “angry” a mid-rising tone). Infants born into tone languages (a term which includes “pitch accent” languages; Hyman, 2009) are faced with a challenge of discovering how pitch patterns in the ambient language distinguish different word meanings—hence, they must *disaggregate* pitch in the input into non-referential and potentially referential information. Infants learning a (non-tone) lexical stress language must discover that pitch has no direct, but only indirect referential significance as one of the cues associated with stress (next to other cues, e.g., duration). Detection of the referential significance of pitch poses a critical challenge for infants when they are learning their first words. Yet several studies suggest infants discover the presence/absence of lexical tones before their first birthday. Tone-learning infants retain their initial ability to discriminate tones, while infants exposed to a non-tone language lose it between 6 and 9 months (Mattock and Burnham, 2006; Mattock et al., 2008; Yeung et al., 2013; Liu and Kager, 2014; Götz et al., 2018), before losing the ability to learn tone-to-word associations, which they still possess at 9 months (Yeung et al., 2014), by 18 months (Singh et al., 2014; Hay et al., 2015; Burnham et al., 2018; Liu and Kager, 2018). How are infants able to disaggregate pitch into non-referential affective and referential linguistic information?

Infants' environments are rich in affective content, as infant-directed speech (IDS) is characterized by exaggerated pitch contours reflecting “free vocal expression of emotion” (Trainor et al., 2000), which attracts infants' attention (Cooper and Aslin, 1990; Werker et al., 1994), yet does not a priori facilitate tone acquisition, as it may partially obscure contrastive shapes of tones (Papoušek and Hwang, 1991; Kitamura et al., 2002). Pitch exaggeration in IDS may be partly compensated by tonal hyper-articulation (Liu et al., 2007; Xu Rattanasone et al., 2013; Tang et al., 2017), yet to what extent precisely is an open issue. In order to facilitate disaggregation of referential and affective pitch, young infants may draw on their ability to recognize vocal and visual expression of affect.

The ability to interpret speech prosody as having affective value emerges early in life. Pitch contours in IDS presumably carry innately specified affective meanings to young infants, eliciting attention, arousal, approval, and disapproval (see Fernald, 1992, for a review). Neonates show an increase in eye opening responses to happy vocal stimuli as compared to other expressions (angry, sad, neutral), however only for their native language (Mastropieri and Turkewitz, 1999), suggesting prenatal influence on perception of vocal affect. By 5 months, infants reliably discriminate affect, detecting changes in vocal affect from sad to happy (Walker-Andrews and Grolnick, 1983); 7-month-olds show different ERP responses to affective (happy or angry) vs. neutral prosody (Grossmann et al., 2005). Yet infants' ability to *discriminate* affect may not provide a reliable basis for affective-referential pitch disaggregation; perhaps it should be matched by an ability to *understand* emotion in speech. However, this ability is not developed until 4–5 years (Quam and Swingley, 2012). School-aged children (around age 10) experience difficulties integrating vocal affect with lexical content (Friend, 2000, 2001, 2003; Friend and Bryant, 2000; Morton and Trehub, 2001; Morton et al., 2003). Since the ability to understand emotion in speech develops so slowly, it is worth exploring how affective-referential pitch disaggregation during the first year of life might be supported not only by auditory/vocal cues, but also by visual/facial cues.

By 4–6 months of age, infants in spite of their reduced visual processing can discriminate their native language from other languages partly by relying on visual cues accompanying gestures such as vocalic lip rounding (Weikum et al., 2007). In comparison, visual cues to tonal gestures are weak and unreliable to native listeners (Chen and Massaro, 2008; Hannah et al., 2017). Young infants (4-month-olds) can detect different emotions (happy, angry, sad) when presented with facial-vocal cues (Flom and Bahrick, 2007), an ability emerging prior to affect detection based on unimodal cues (Walker-Andrews, 1997). In light of infants' early sensitivity to facial-vocal cues to affect, the hypothesis can be proposed that affective-referential pitch disaggregation draws on facial affective cues accompanying vocal affect. By labeling pitch information as affective, infants may focus their linguistic attention to residual pitch information that has no clear affective interpretation, which includes referential information.

A neurological marker of affective-referential pitch disaggregation may be obtained in the hemispheric specialization for linguistic and affective pitch. A functional asymmetry between the right hemisphere (RH; dominant in processing pitch changes and emotional vocalization) and the left hemisphere (LH; dominant in processing speech, in particular segmental information) occurs in neonates (Dehaene-Lambertz, 2000; Peña et al., 2003). Native listeners process linguistically relevant lexical pitch dominantly in LH (Wang et al., 2001); affective pitch dominantly in RH (Edmondson et al., 1987). Yet hemispheric lateralization of linguistic and affective pitch processing remains a controversial issue (Wong, 2002; Zatorre and Gandour, 2008). Turning to infant studies, early RH specialization for pitch processing is found in neonates (Arimitsu et al., 2011); 3-month-old Japanese infants show stronger RH responses to natural speech, which includes pitch contours, as compared to prosodically flattened speech (Homae et al., 2006). The processing of lexical pitch is lateralized to LH in Japanese infants between 4 and 10 months (Sato et al., 2010; see Minagawa-Kawai et al., 2011 for discussion). Plausibly, the disaggregation of affective-referential pitch involves a functional specialization of the brain's hemispheres: general (affective and linguistic) pitch processing starts out in RH, while disaggregation amounts to a lateralization of linguistic pitch processing to LH. Infants' detection of affect, guided by vocal-facial cues, provides the key ability. A second hypothesis is proposed to this effect: the more emotional speech is, the more dominant RH becomes in speech processing; conversely, less emotional speech implies a decreased role for RH in pitch processing, enabling a partial shift of pitch processing to LH, the dominant hemisphere for speech processing. This predicts that (the perceived amount of) facial affect influences the locus of pitch processing in the infant brain.

In sum, affective-referential pitch disaggregation by infants may be accomplished by a combination of two (possibly innate) abilities, matching the two hypotheses stated above: (a) recognition of affect in pitch contours and integration of audiovisual (vocal-facial) cues on affect; (b) hemispheric specialization for pitch processing, where RH acts as “emotion attractor” and LH as “language attractor.” Integrating research on early tone perception, audiovisual affect recognition, and hemispheric specialization may open a new perspective on how infants manage to detect the presence/absence of lexical tone in their native language.

## Author contributions

The author confirms being the sole contributor of this work and has approved it for publication.

### Conflict of interest statement

The author declares that the research was conducted in the absence of any commercial or financial relationships that could be construed as a potential conflict of interest.
